# Clonal analysis of immunodominance and cross-reactivity of the CD4 T cell response to SARS-CoV-2

**DOI:** 10.1126/science.abg8985

**Published:** 2021-05-18

**Authors:** Jun Siong Low, Daniela Vaqueirinho, Federico Mele, Mathilde Foglierini, Josipa Jerak, Michela Perotti, David Jarrossay, Sandra Jovic, Laurent Perez, Rosalia Cacciatore, Tatiana Terrot, Alessandra Franzetti Pellanda, Maira Biggiogero, Christian Garzoni, Paolo Ferrari, Alessandro Ceschi, Antonio Lanzavecchia, Federica Sallusto, Antonino Cassotta

**Affiliations:** 1 Institute for Research in Biomedicine, Università della Svizzera Italiana, 6500 Bellinzona, Switzerland.; 2 Laboratory of Immunogenetics, Department of Transfusion Medicine and Immuno-Hematology, Fondazione I.R.C.C.S. Policlinico S. Matteo, 27100 Pavia, Italy.; 3 Clinical Trial Unit, Ente Ospedaliero Cantonale, 6500 Bellinzona, Switzerland.; 4 Clinic of Internal Medicine and Infectious Diseases, Clinica Luganese Moncucco, 6900 Lugano, Switzerland.; 5 Faculty of Biomedical Sciences, Università della Svizzera italiana, 6900 Lugano, Switzerland.; 6 Department of Internal Medicine, Ente Ospedaliero Cantonale, 6500 Bellinzona, Switzerland.; 7 Prince of Wales Hospital Clinical School, University of New South Wales, Sydney, New South Wales 2052, Australia.; 8 Division of Clinical Pharmacology and Toxicology, Institute of Pharmacological Sciences of Southern Switzerland, Ente Ospedaliero Cantonale, 6900 Lugano, Switzerland.; 9 Department of Clinical Pharmacology and Toxicology, University Hospital Zurich, 8091 Zurich, Switzerland.; 10 National Institute of Molecular Genetics, 20122 Milano, Italy.; 11 Institute of Microbiology, ETH Zürich, 8093 Zurich, Switzerland.

## Abstract

The identification of CD4^+^ T cell epitopes is instrumental for the design of subunit vaccines for broad protection against coronaviruses. Here we demonstrate in COVID-19-recovered individuals a robust CD4^+^ T cell response to naturally processed SARS-CoV-2 spike (S) and nucleoprotein (N), including effector, helper, and memory T cells. By characterizing 2943 S-reactive T cell clones from 34 individuals, we found that 34% of clones and 93% of individuals recognized a conserved immunodominant S346-365 region within the RBD comprising nested HLA-DR- and HLA-DP-restricted epitopes. Using pre- and post-COVID-19 samples and S proteins from endemic coronaviruses, we identify cross-reactive T cells targeting multiple S protein sites. The immunodominant and cross-reactive epitopes identified can inform vaccination strategies to counteract emerging SARS-CoV-2 variants.

The identification of T cell epitopes in disease causing organisms is a challenge in view of the polymorphism of HLA class II molecules and the variability of rapidly mutating pathogens. In the context of the COVID-19 pandemic, bioinformatic analysis (*1*) has been used to predict T cell epitopes in SARS-CoV-2 proteins (*2*, *3*) and to produce peptide pools to stimulate peripheral blood mononuclear cells (PBMCs) and enumerate antigen-specific T cells. These studies revealed a robust CD4^+^ and CD8^+^ T cell response against SARS-CoV-2 proteins in recovered patients (*2*–*6*) and a level of cross-reactivity with endemic coronaviruses in pre-pandemic samples (*7*–*9*).

A limitation of bioinformatic predictions is the difficulty in identifying immunodominant epitopes, since immunodominance is determined by multiple factors such as antigen processing, T cell repertoire, HLA alleles, and preexisting cross-reactive immunity (*10*–*12*). To identify naturally processed immunodominant CD4^+^ T cell epitopes, we took the unbiased approach of stimulating memory T cells with protein-pulsed antigen presenting cells (APCs), followed by the isolation of T cell clones to precisely map the epitope recognized (*13*).

PBMCs from a first cohort of 14 patients who had recovered from mild-to-severe COVID-19 (table S1) were used to isolate total CD4^+^ T memory cells or T central memory (Tcm), T effector memory (Tem), and circulating T follicular helper (cTfh) cells (fig. S1A). The cells were labeled with CFSE and stimulated with autologous monocytes in the presence of recombinant SARS-CoV-2 spike (S) or nucleoprotein (N). In all individuals, we observed a strong response to both antigens in terms of proliferation and IFN-γ production (Fig. 1, A and B, and fig. S1, B and C). Proliferating T cells were detected at different levels in Tcm, Tem, and cTfh, consistent with a recent report (*14*), and over a one-year period (fig. S1D). By contrast, the CD4^+^ memory T cell response to SARS-CoV-2 proteins in unexposed individuals was low or undetectable (Fig. 1B and fig. S1C), consistent with the presence of a few cross-reactive T cells primed by endemic coronaviruses (*4*, *5*, *9*).

**Fig. 1:**
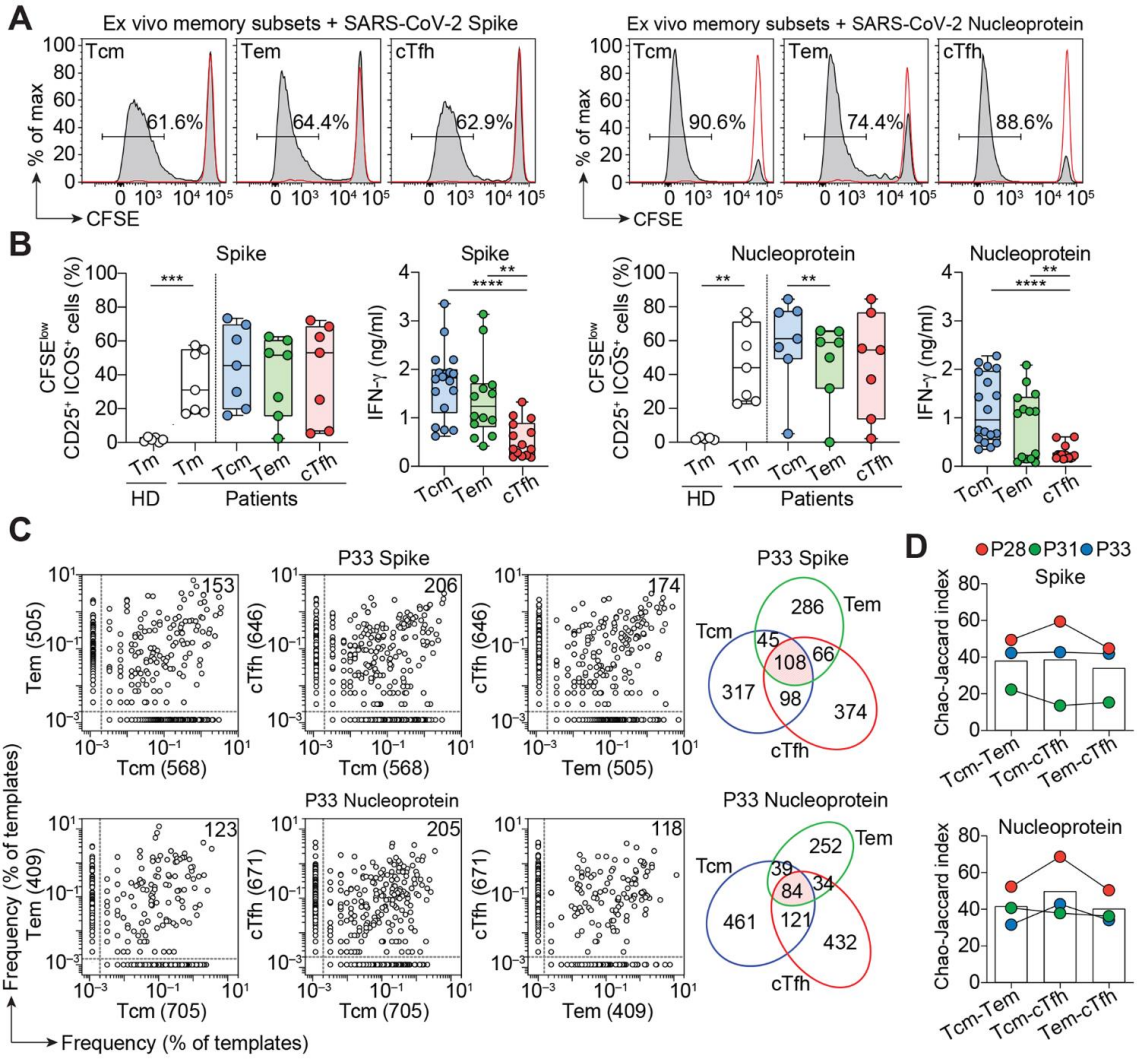
Robust T cell response to SARS-CoV-2 S and N in CD4^+^ memory T cell subsets. Total CD4^+^ memory T cells from seven COVID-19 recovered patients and six unexposed (pre-COVID-19) healthy donors (HD) or CD4^+^ Tcm, Tem and cTfh cells from seven COVID-19 recovered patients were labeled with CFSE and cultured with autologous monocytes in the presence or absence of recombinant SARS-CoV-2 S or N. (**A**) CFSE profiles on day 7 and percentage of CFSE^lo^ proliferating Tcm, Tem, and cTfh cells in a representative recovered patient. Negative controls of T cells cultured with monocytes in the absence of antigen are shown as red lines. (**B**) Individual values, median and quartile values of percentage of CFSE^lo^CD25^+^ICOS^+^ cells in total CD4^+^ memory and CD4^+^ Tcm, Tem and cTfh subsets in recovered patients and healthy donors. Shown are also IFN-γ concentrations in the day 7 culture supernatants of SARS-CoV-2 S- or N-stimulated memory T cell subsets. IFN-γ concentrations were below detection limit in HD and in negative control cultures. ****, *P*-value < 0.0001 ***, *P*-value < 0.001 **, *P*-value < 0.01 as determined by two-tailed unpaired *t* test (total CD4^+^ memory and IFN-γ) or by two-tailed paired *t* test (CD4^+^ Tcm, Tem, and cTfh). (**C**) Pairwise comparison of TCR Vβ clonotype frequency distribution in samples of T cells isolated from S-stimulated Tcm, Tem, or cTfh subsets (initial input 5×10^5^ cells per subset) from P33. Frequencies are shown as percentage of productive templates. The total number of clonotypes is indicated in the *x*- and *y*-axes. Values in the upper right corner represent the number of clonotypes shared between two samples. The Venn diagrams show the number of unique and shared clonotypes between Tcm, Tem and cTfh subsets. (**D**) The bar histograms show the Chao–Jaccard similarity index between pairs of TCR Vβ repertoires in three donors.

The clonal composition of SARS-CoV-2-reactive T cells and the relationship between different memory subsets was studied in three individuals (P28, P31, and P33) by TCR Vβ sequencing. Tcm, Tem, and cTfh cell lines comprised on average 908, 480, and 697 S-reactive clonotypes and 1452, 623, and 908 N-reactive clonotypes, respectively (Fig. 1C and fig. S2). Interestingly, several of the most expanded clonotypes were shared between two and even among all three subsets (Fig. 1, C and D), indicating a polyfunctional response consistent with previous studies on intraclonal diversification of antigen-primed CD4^+^ T cells (*15*, *16*).

In view of the interest for the design of a subunit vaccine, we analyzed in depth the CD4^+^ T cell response to the S protein and in particular to the RBD, which is the main target of neutralizing antibodies (*17*, *18*). CD4^+^ T cells from a larger cohort of 34 COVID-19 individuals (table S1) were stimulated with S protein-pulsed monocytes and proliferating T cells were cloned by limiting dilution. We obtained 2943 T cell clones and mapped their specificity using three pools of peptides spanning S1_ΔRBD_, RBD, and S2 (Fig. 2, A and B). RBD-specific T cell clones were found in 32 out of 34 donors, accounting on average for 20% of the response to the S protein (Fig. 2B). Using a matrix-based approach, we mapped the epitope specificity of 1254 RBD-reactive CD4^+^ T cell clones (Fig. 2C) and found that in each individual the clones recognize multiple sites that collectively spanned almost all the RBD sequence. However, certain regions emerged as immunodominant, such as those spanning residues S346-385 and S446-485. Strikingly, a 20-amino-acid region (S346-365) was recognized by 94% of the individuals (30 out of 32) and by 33% of the clones (408 out of 1254) (Fig. 2D). This region is highly conserved among human Sarbecoviruses, including the recently emerged variants of concern (VOC), and zoonotic Sarbecoviruses (Fig. 2E) (*19*). RBD- and S346-365-specific T cell clones were found in different memory subsets of COVID-19 individuals and were also isolated from individuals following SARS-CoV-2 mRNA vaccination (fig. S3). Thus, RBD is highly immunogenic in vivo and contains a large number of naturally processed T cell epitopes including a conserved immunodominant region.

**Fig. 2:**
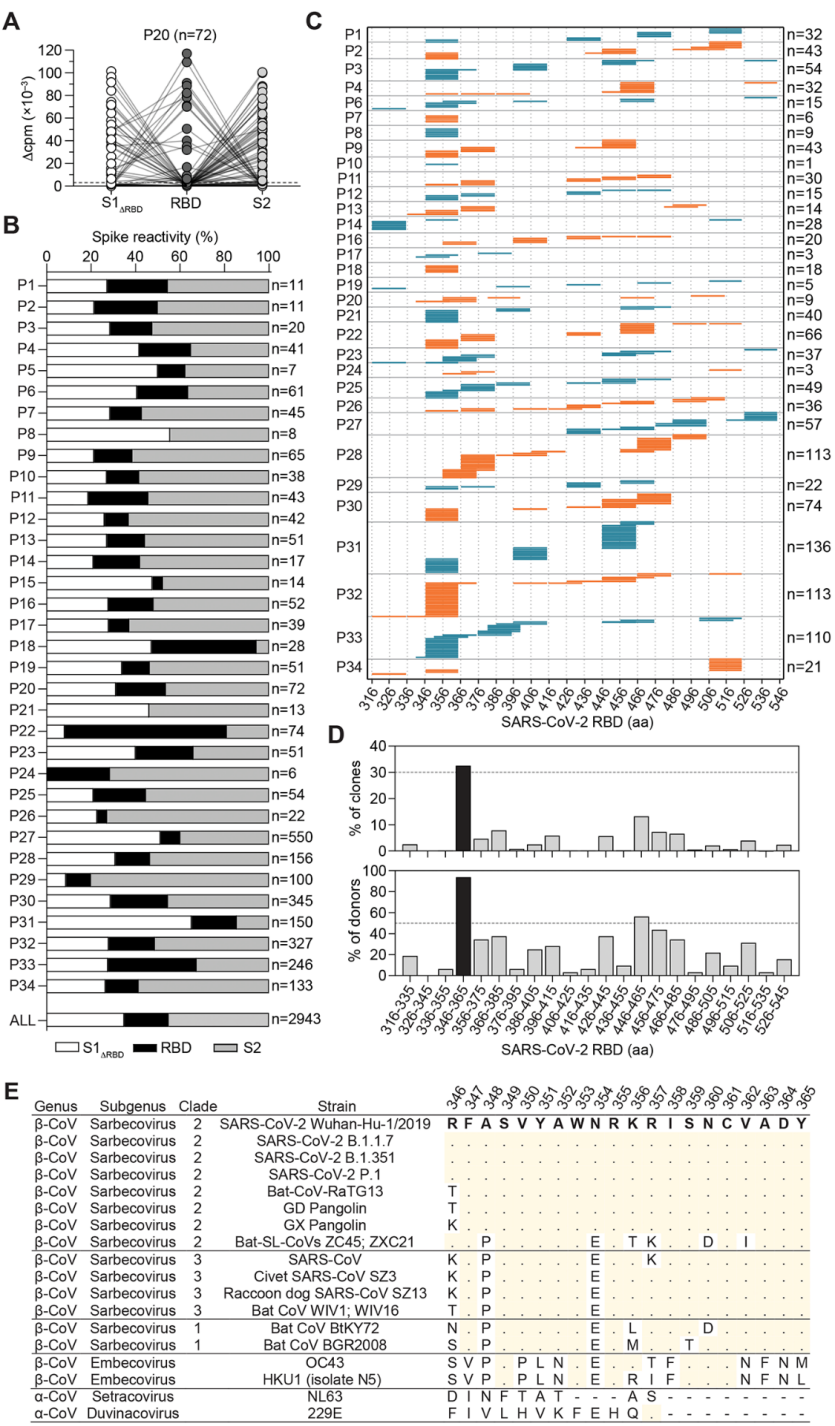
CD4^+^ T cell clones target multiple sites on the S protein. (**A** and **B**) CD4^+^ T cell clones (n=2943) were isolated from S-reactive cultures of 34 COVID-19 individuals and their specificity was mapped by stimulation with autologous B cells and three pools of 15-mer peptides overlapping of 10 spanning the S1-325 and S536-685 sequences (S1_ΔRBD_, 91 peptides), the S316-545 sequence (RBD, 44 peptides), and the S676-1273 sequence (S2, 118 peptides), using as readout ^3^H-thymidine incorporation. (A) Characterization of representative T cell clones (n = 72) from P20. Proliferation was assessed on day 3 after a 16-hours pulse with ^3^H-thymidine and expressed as counts per min after subtraction of the unstimulated control value (Δcpm). (B) Percentage of T cell clones specific for S1_ΔRBD_ (white), RBD (black), and S2 (gray) in the 34 individuals tested. The number of clones tested is indicated on the right. The distribution of all S-reactive T cell clones isolated from all 34 individuals (ALL, n=2943) is also indicated. (**C**) RBD-specific T cell clones (n=1254) isolated from 32 individuals were further characterized for their epitope specificity using 15-mer peptides overlapping by 10 spanning the S316-545 RBD sequence. The 20-mer specificity of each clone is represented by a horizontal line and the total number of clones mapped for each individual is indicated on the right. (**D**) Percentage of clones specific and percentage of individuals carrying T cells specific for different 20-mer segments of the RBD. Data for the immunodominant region S346-365 is shown in black. (**E**) Sequence alignments of the SARS-CoV-2 immunodominant region S346-365 with homologous sequences in different Sarbecoviruses, human and animal SARS-related coronaviruses, and alpha and beta coronaviruses. Dots indicate identity to SARS-CoV-2 reference strain; dashes indicate deletions.

To dissect the CD4^+^ T cell response to the immunodominant S346-365 region, we sequenced TCR Vβ chains of 329 specific T cell clones. The 206 clonotypes identified used a broad spectrum of TCR Vβ genes and, even in the same individual, carried different CDR3 sequences (Fig. 3, A and B, and table S2). In P31 and P33, certain S346-365 clonotypes were detected in ex vivo memory T cells among the top 5% (Fig. 3C). Using blocking antibodies we determined that most of the T cell clones analyzed (n=247 from 22 individuals) were HLA-DR-restricted, whereas the remaining (n=50 from five individuals) were HLA-DP- and one was HLA-DQ-restricted (Fig. 3, D and E). Using truncated peptides and T cell clones from individuals with different HLA type (table S3), we defined two HLA-DR-restricted epitopes (VYAWNRKRIS and RFASVYAWNRKR), and one HLA-DP-restricted epitope (NRKRISNCVAD) (Fig. 3F). Thus, the S346-365 region comprises at least three nested epitopes recognized in association with different allelic forms of HLA-DR or HLA-DP by T cell clones that use a large set of TCR Vβ genes and CDR3 of different sequence and length.

**Fig. 3:**
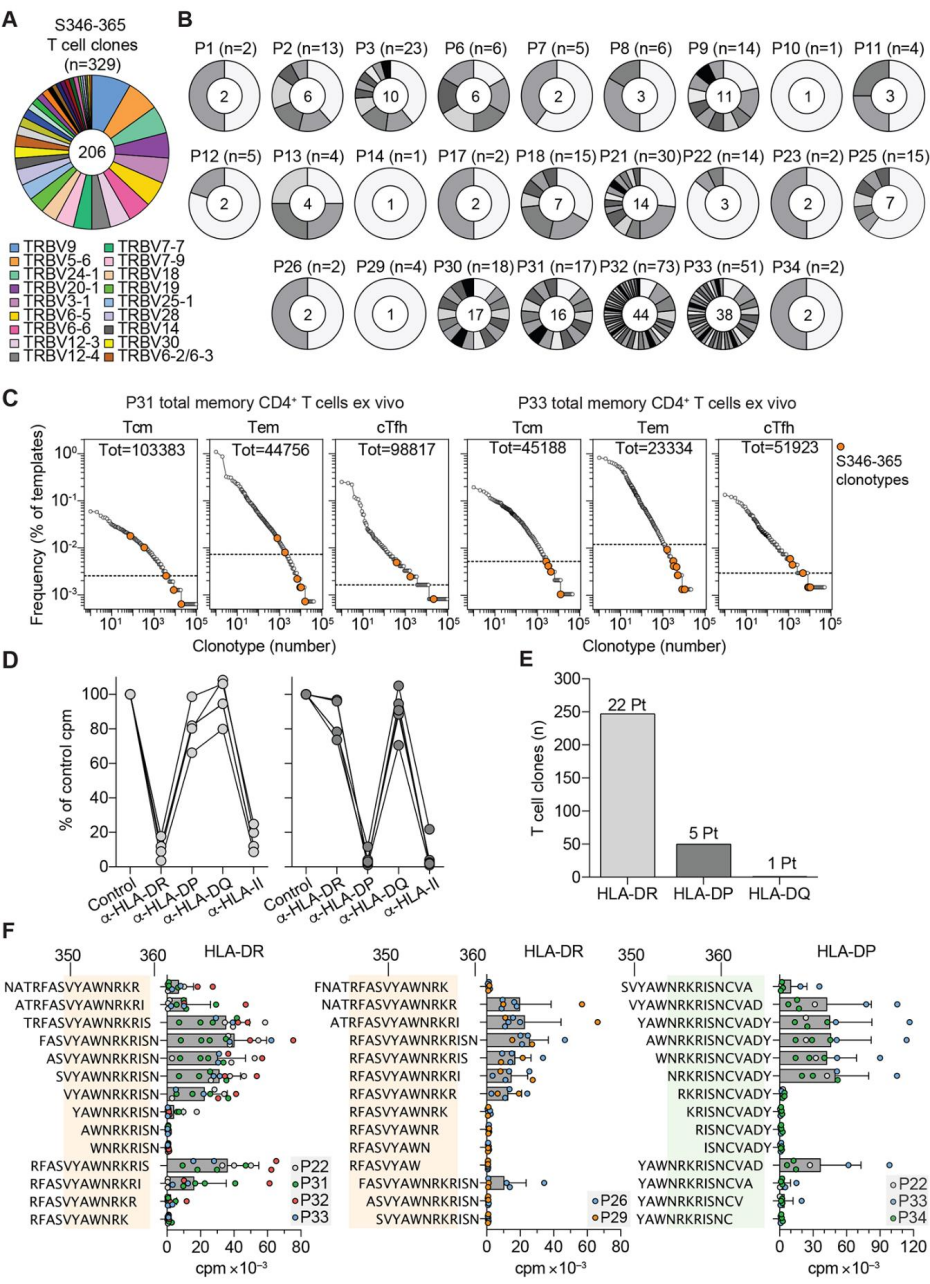
The immunodominant S346-365 RBD region contains nested epitopes targeted by a diverse repertoire of T cells restricted by HLA-DR and HLA-DP. (**A** and **B**) Rearranged TCR Vβ sequences of S346-365-reactive CD4^+^ T cell clones (n=329) isolated from 25 COVID-19-recovered individuals as determined by RT-PCR and Sanger sequencing. (A) TCR Vβ gene usage of the 206 unique clonotypes. Slices in the chart represent different Vβ genes and their size is proportional to the number of clonotypes using that particular gene. Color-coded legend is reported for the top 18 Vβ genes (used by at least five different TCR Vβ clonotypes). (B) Number of S346-365-reactive T cell clones and clonotypes identified in the 25 individuals. Slices in the charts represent different TCR Vβ clonotypes and their size is proportional to the number of sister clones bearing the same sequence. The number of clones is reported on top and the number of clonotypes at the center of the pie chart. (**C**) Frequency distribution of TCR Vβ clonotypes from CD4^+^ Tcm Tem and cTfh subsets sequenced directly after ex vivo isolation from P31 and P33. Colored circles mark the TCR Vβ clonotypes found among the S346-365-specific T cell clones isolated from the same individual. Dotted lines in the graphs indicate the frequency threshold of the top 5% expanded clonotypes. (**D**) HLA class II isotype restriction of S346-365-specific T cell clones (n=10) isolated from P33 as determined by stimulation with peptide-pulsed autologous APCs in the absence (control) or in the presence of blocking antibodies to HLA-DR, -DP, -DQ or pan HLA class II. Proliferation was assessed on day 3 after a 16-hour pulse with ^3^H-thymidine. Data are expressed as percentage of control counts per minute (cpm). (**E**) HLA class II isotype usage by S346-365-reactive CD4^+^ T cell clones (n=298) from 24 individuals, as determined by >80% inhibition of proliferation. (**F**) Identification of the minimal peptide recognized by HLA-DR or HLA-DP-restricted S346-365-reactive CD4^+^ T cell clones (n=23) isolated from seven individuals, as determined by stimulation with autologous APCs pulsed with a panel of truncated peptides. Proliferation was assessed on day 3 and expressed as counts per minute (cpm). Bars indicate mean + SD; circles indicate individual clones. The minimal amino acid sequences recognized by T cell clones are highlighted with colored shading.

To address the extent of T cell cross-reactivity between different S proteins, SARS-CoV-2 S-specific T cell lines from P28 and P33 were relabeled with CFSE and stimulated with S proteins from endemic human coronaviruses. In the secondary cultures, a robust proliferation was observed in response to SARS-CoV and HKU1 (Fig. 4A). Interestingly, a sizeable fraction of SARS-CoV-2 clonotypes (ranging from 7 to 25%) were found in SARS-CoV and/or HKU1 secondary cultures, consistent with a substantial degree of T cell crossreactivity (fig. S4). To corroborate this finding, we isolated from secondary cultures several clones that proliferated in response to two or even three different naturally processed S proteins (Fig. 4B and table S4).

**Fig. 4:**
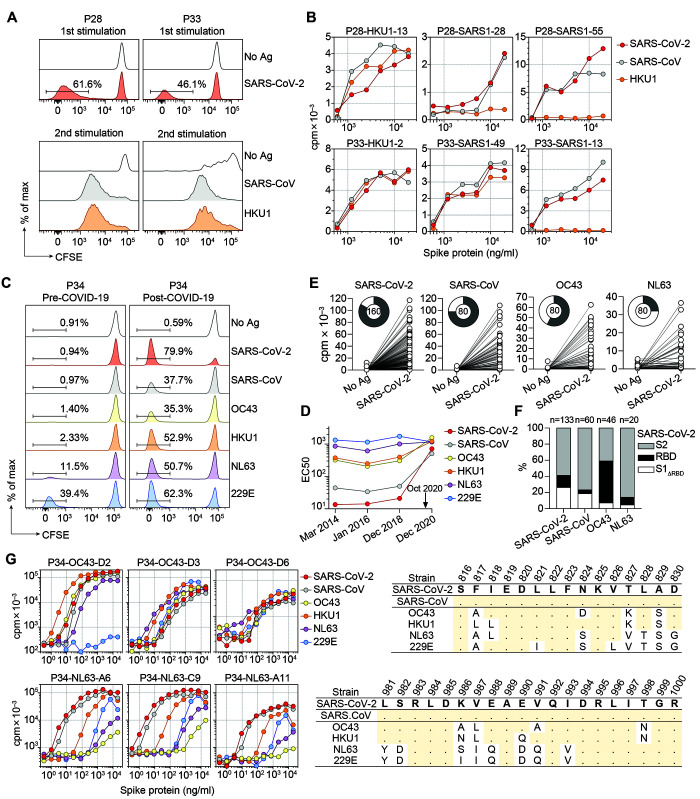
Identification of coronavirus S protein cross-reactive T cell clones. (**A** and **B**) CFSE-labeled CD4^+^ memory T cells from P28 and P33 were stimulated with recombinant SARS-CoV-2 S protein in the presence of autologous monocytes. CFSE^lo^ cells were expanded with IL-2 for 10 days and relabeled and restimulated with S protein from human beta (SARS-CoV, HKU1, and OC43) or alpha (NL63 and 229E) coronaviruses. T cell clones from proliferating cultures were isolated and tested for cross-reactivity against different S proteins. (A) CFSE profiles from primary and secondary stimulation in the absence or in the presence of the indicated antigens. (B) Proliferative response (day 3 cpm) of representative T cell clones isolated from secondary cultures to autologous APCs pulsed with titrated doses of recombinant S proteins from SARS-CoV-2, SARS-CoV, or HKU1. (**C** to **G**) Multiple blood samples were obtained from donor P34 several years before and 1.5 months after COVID-19 and characterized as far as memory T cells and serum antibody levels. (C) T cell proliferation (CFSE dilution) in pre-COVID-19 (2018) and post-COVID-19 samples in response to autologous monocytes pulsed with different S proteins. (D) Time course of serum IgG antibodies against different coronavirus S proteins as determined by ELISA (EC50 values). These data demonstrate that together with a strong induction of serum antibodies to SARS-CoV-2, antibody titers against HKU1 and OC43 also increased in the post-COVID-19 sample. (E) Proliferative response to a pool of SARS-CoV-2 peptides of T cell clones obtained from post-COVID-19 CFSE^lo^ cultures stimulated by SARS-CoV-2, SARS-CoV, OC43 or NL63. Pie charts show the total number of clones tested and the fraction of responsive clones. (F) Reactivity of T cell clones isolated from each culture (in E) was further mapped by stimulation with pools of peptides spanning the S1_ΔRBD_, RBD, and S2 regions of SARS-CoV-2 S protein. The histograms show the percentage of clones specific for each region. Total number of clones tested is indicated on top. (G) Characterization of cross-reactive T cell clones specific for S proteins isolated from P34 post-COVID-19 sample. The peptides recognized are indicated on the right panels. Shown are sequence alignment of the recognized SARS-CoV-2 epitopes (S816-830 and S981-1000) with homologous sequences of endemic alpha and beta coronaviruses. Dots indicate identity to SARS-CoV-2 reference strain.

Crossreactive T cells may derive from preexisting memory cells or from the priming of naïve T cells. We therefore analyzed a COVID-19-recovered individual from whom we had previously cryopreserved PBMCs. A robust memory CD4^+^ T cell proliferation in the pre-COVID-19 sample was detected against NL63 and 229E S proteins, whereas the response to HKU1 and OC43 was limited and the response to SARS-CoV and SARS-CoV-2 undetectable (Fig. 4C). Conversely, in the post-COVID-19 sample, strong T cell proliferation was observed not only in response to SARS-CoV-2, but also in response to all other alpha and beta coronavirus S proteins (Fig. 4, C and D) and shared clonotypes were detected between SARS-CoV-2 and endemic coronavirus S protein-stimulated cultures (fig. S5A). Furthermore, T cell clones isolated from cultures stimulated by SARS-CoV, OC43, or NL63 proliferated in response to the SARS-CoV-2 S peptide pool and their specificity was mapped primarily to the S2 region (Fig. 4, E and F), consistent with its high degree of sequence conservation (*20*–*22*). Notably, T cell clones that fully cross-reacted with all S proteins mapped to the highly conserved fusion peptide (Fig. 4G).

To ask whether S-reactive T cells in the post-COVID-19 sample could be detected in pre-pandemic samples, we performed clonotypic analysis of total memory T cells on the post-COVID-19 sample and on samples collected in 2014 and 2017. Most of the SARS-CoV-2 reactive clonotypes identified above were found only in the post-COVID-19 sample, consistent with priming of naïve T cells (fig. S5B). By contrast, clonotypes reactive to endemic coronaviruses were found at comparable number at all time points. Notably, T cell clonotypes against the highly conserved fusion peptide could be tracked back to the 2014 sample and found to be expanded in the post-COVID-19 sample (fig. S5C). These findings demonstrate that pre-existing crossreactive memory T cells are recalled and expanded upon SARS-CoV-2 infection.

The robust CD4^+^ T cell response to the RBD and the identification of the S346-365 immunodominant region conserved in the emerging SARS-CoV-2 VOC provide the rationale for the development of a subunit vaccine based on RBD, since this is the target of most neutralizing antibodies (*17*, *18*). These findings were not anticipated in previous studies based on bioinformatics predictions (*2*, *3*) and short-term peptide stimulation of PBMCs, highlighting the value of combining T cell stimulation with protein antigens with cloning and TCR sequencing for the dissection of antigen-specific T cell repertoires.

The immunodominance of RBD S346-365 at the individual and at the population level may be due to the presence of three nested T cell epitopes presented by HLA-DR and HLA-DP and to the relative abundance of naturally processed peptides, as recently reported by immunopeptidomics (*23*). Interestingly, the S346-365 region is also a contact site for the broadly reactive neutralizing antibody S309 (*24*), providing a striking example of convergence of B and T cells around a conserved epitope.

Our study provides also evidence for the recall of preexisting cross-reactive memory T cells upon SARS-CoV-2 infection. However, this phenomenon reminiscent of the “original antigenic sin” (*25*) does not prevent a robust and persistent primary response to new epitopes of SARS-CoV-2 that is characterized by extensive intraclonal diversification into Tem, cTfh and Tcm, which represent inflammatory, helper and long-lived memory T cells (*26*, *27*). The availability of a large number of cross-reactive T cell clones is not only instrumental to defining target sites in relevant pathogens but also to understanding whether cross-reactivity is due to epitope structural similarities or to TCR binding degeneracy (*11*, *28*).

The possibility of leveraging a robust cross-reactive T helper cell function against conserved sites will be instrumental to drive neutralizing antibody responses to adaptive vaccines that incorporate escape mutations found in emerging SARS-CoV-2 variants.

## Supplementary Materials

science.sciencemag.org/cgi/content/full/science.abg8985/DC1 Materials and Methods Tables S1 to S5 Figs. S1 to S5 References (*29*, *30*) MDAR Reproducibility Checklist View/request a protocol for this paper from *Bio-protocol*.
